# Expression of 3-Methylcrotonyl-CoA Carboxylase in Brain Tumors and Capability to Catabolize Leucine by Human Neural Cancer Cells

**DOI:** 10.3390/cancers14030585

**Published:** 2022-01-24

**Authors:** Eduard Gondáš, Alžbeta Kráľová Trančíková, Eva Baranovičová, Jakub Šofranko, Jozef Hatok, Bhavani S. Kowtharapu, Tomáš Galanda, Dušan Dobrota, Peter Kubatka, Dietrich Busselberg, Radovan Murín

**Affiliations:** 1 Department of Medical Biochemistry, Jessenius Faculty of Medicine in Martin, Comenius University in Bratislava, Malá Hora 4D, 036 01 Martin, Slovakia; gondas3@uniba.sk (E.G.); sofranko4@uniba.sk (J.Š.); jozef.hatok@uniba.sk (J.H.); dusan.dobrota@uniba.sk (D.D.); peter.kubatka@uniba.sk (P.K.); 2 Biomedical Center Martin-BioMed, Jessenius Faculty of Medicine in Martin, Comenius University in Bratislava, 036 01 Martin, Slovakia; alzbeta.trancikova@uniba.sk (A.K.T.); eva.baranovicova@uniba.sk (E.B.); 3 Institute of Neuroimmunology, Slovak Academy of Sciences, 845 10 Bratislava, Slovakia; 4 Prof. Brien Holden Eye Research Center, L V Prasad Eye Institute, Champalimaud Translational Centre for Eye Research Kallam Anji Reddy Campus, Hyderabad 500034, India; bhavani.k@lvpei.org; 5 Department of Neurosurgery, Slovak Medical University, Roosevelt Hospital, 975 17 Banska Bystrica, Slovakia; tgalanda@imafexbb.sk; 6 Department of Physiology and Biophysics, Weill Cornell Medicine in Qatar, Education City, Qatar Foundation, Doha 24144, Qatar; dib2015@qatar-med.cornell.edu

**Keywords:** cancer cells, metabolism, leucine, branched-chain amino acid, 3-methylcrotonyl-CoA carboxylase, ketone bodies, citrate, acetyl-CoA

## Abstract

**Simple Summary:**

Leucine is a ketogenic amino acid that is essential for sustaining cellular metabolism. To understand the leucine catabolizing capability of brain cancer cells, in this study, we evaluated the leucine removing ability of human glioma, glioblastoma, and neuroblastoma cells from their culture media. In addition, we also studied the generation of 2-oxoisocaproate, ketone bodies, and citrate. Further, we applied immunoprobing methods to evaluate the expression of 3-methylcrotonyl-CoA carboxylase (MCC) in cultured cells, and also in the human glioblastoma, astrocytoma, oligodendroglioma, and meningioma forming cells. Our results show that human cancer cells, in culture or in situ, express MCC and catabolize leucine. These results indicate that brain cancer cells could employ leucine catabolites as a substrate for their metabolism.

**Abstract:**

Leucine is an essential, ketogenic amino acid with proteinogenic, metabolic, and signaling roles. It is readily imported from the bloodstream into the brain parenchyma. Therefore, it could serve as a putative substrate that is complementing glucose for sustaining the metabolic needs of brain tumor cells. Here, we investigated the ability of cultured human cancer cells to metabolize leucine. Indeed, cancer cells dispose of leucine from their environment and enrich their media with the metabolite 2-oxoisocaproate. The enrichment of the culture media with a high level of leucine stimulated the production of 3-hydroxybutyrate. When ^13^C_6_-leucine was offered, it led to an increased appearance of the heavier citrate isotope with a molar mass greater by two units in the culture media. The expression of 3-methylcrotonyl-CoA carboxylase (MCC), an enzyme characteristic for the irreversible part of the leucine catabolic pathway, was detected in cultured cancer cells and human tumor samples by immunoprobing methods. Our results demonstrate that these cancer cells can catabolize leucine and furnish its carbon atoms into the tricarboxylic acid (TCA) cycle. Furthermore, the release of 3-hydroxybutyrate and citrate by cancer cells suggests their capability to exchange these metabolites with their milieu and the capability to participate in their metabolism. This indicates that leucine could be an additional substrate for cancer cell metabolism in the brain parenchyma. In this way, leucine could potentially contribute to the synthesis of metabolites such as lipids, which require the withdrawal of citrate from the TCA cycle.

## 1. Introduction

Selective growth and proliferation are considered as the typical features of cancer cells [1,2,3]. These two cellular properties correlate with each other by altering and adjusting the cancer metabolism, by reprogramming anabolic and catabolic pathways through modulated gene expression [4]. However, the transformed metabolism of cancer cells depends on the availability of appropriate substrates that should be taken up from their milieu [5]. In addition to glucose, essential amino acids are also indispensable molecules for sustaining the cellular metabolism of cancer cells [6,7,8]. Among the essential amino acids, a group of branched-chain amino acids (BCAA) possesses several roles in metabolism [9] and proliferation of cancer cells [10,11] with an impact on tumorigenesis [12,13].

Leucine, isoleucine, and valine are essential amino acids that constitute the group of BCAA. The prime metabolism of BCAA, which includes the transmembrane transport and two-step conversion to their related acyl-CoA derivatives, is facilitated by several common transporters and enzymes. Cancer cells overexpress the transporters for BCAA import [14,15,16] from their microenvironments and mediate intracellular accumulation of BCAA [12]. The cells utilize the BCAAs in several ways [6,7,8,9,16,17,18,19], due to their capability of donating nitrogen [11] and carbon atoms into the cellular metabolism [10,20,21]. The prime reaction in the metabolism of BCAAs is their reversible transamination with 2-oxoglutarate to their cognate branched-chain 2-keto acids (BCKA) and glutamate that is catalyzed by branched-chain aminotransferase (BCAT). Subsequently, BCKAs can enter the irreversible part of the catabolism by oxidative decarboxylation catalyzed by BCKA dehydrogenase (BCKDH) complex to corresponding branched-chain acyl-CoA derivatives. The catabolism of ketogenic leucine yields acetoacetate and acetyl-CoA, while glucogenic valine is converted into propionyl-CoA. Since the carbon atoms of isoleucine could enter the intermediary metabolism as acetyl-CoA and propionyl-CoA, isoleucine is both glucogenic and ketogenic [22].

In human cells, two isoforms of BCAT are expressed with specific subcellular localization either in cytosol (BCAT1) or mitochondria (BCAT2). The overexpression of both BCAT isoforms was reported in a cell-type-specific manner among several cancer types [23]. In addition, cultured rat glioblastoma and human cancer cell lines [20] express BCKDH and possess the capability to oxidatively decarboxylate the carbon skeleton of leucine [24]. BCATs and BCKDH are specific for all three BCAAs and BCKAs, respectively. Subsequent conversion of isovaleryl-CoA to acetoacetate and acetyl-CoA requires a distinctive set of enzymes. Those enzymes possess various degrees of specificity against the intermediates of BCAA catabolism, and some of them could also catalyze the conversion of the intermediates from several other metabolic pathways. Among them, 3-methylcrotonyl-CoA carboxylase (MCC) is an enzyme highly specific for carboxylation of 3-methylcrotonyl-CoA [25], which is a typical intermediate from the irreversible part of the leucine catabolic pathway. Due to its high specificity, MCC is considered as an enzymatic marker to identify the leucine catabolizing cells [26,27].

MCC is a mitochondrial, biotin-containing carboxylase composed of two subunits [25]. It is highly expressed in liver and kidney. The mutations in MCC subunit genes lead to MCC deficiency (MIM# 210200 and MIM# 210210), an autosomal recessive disorder with a very variable phenotype [28]. The increased expression of MCC promotes proliferation of prostate [29], breast [30], and colorectal cancer cells [31] and is critical for the oncogenesis of hepatocellular carcinoma [32]. 

The catabolism of leucine produces acetyl-CoA and acetoacetate. Acetoacetate is one of the three ketone bodies and could be either spontaneously decarboxylated to acetone or enzymatically converted to 3-hydroxybutyrate or two molecules of acetyl-CoA. Ketone bodies [33] are considered to enter the metabolism of cancer cells and encourage their capacity to synthesize fatty acids. Indeed, leucine, in addition to its proteinogenic role, is also capable of affecting the growth of pancreatic [21] and breast cancer cells [34] by its impact on their metabolism of fatty acids and lipids. In this respect, leucine’s catabolism could be considered as the endogenous source of the acetate moieties, which could support the lipogenesis in cancer cells [33,35,36]. 

To evaluate the hypothesis that the human cancer cells could use leucine as an alternative substrate for their intermediate metabolism and might use leucine-derived carbon skeleton as a putative source of acetyl-CoA, we evaluated the capability of cultured human astrocytoma, glioblastoma, and neuroblastoma cells to metabolize leucine by ^1^H-NMR and LC-MS methods. In addition, we investigated the presence of MCC among the cultured cancer cells, protein lysates derived from the human brain and prostatic tumors by immuno-detection methods. 

## 2. Materials and Methods

All chemicals, namely leucine, l-[^13^C_6_, ^15^N]leucine, sodium 4-methyl-2-oxovalerate, sodium 2-oxoisocaproate, and citric acid, were purchased from (Sigma St. Louis, MO, USA). HPLC-grade water, acetonitrile, and ammonium acetate were purchased from Sigma. Both antibodies, rabbit anti-MCC [26,27], and human anti-pyruvate dehydrogenase (PDH) [27,37], were a gift from Prof. Dr. Bernd Hamprecht (IFIB, University of Tuebingen, Tübingen, Germany).

### 2.1. Cell Cultures

For the study, we used human SW1088 glioma (ATCC-HTB-12), A172 glioblastoma (ATCC-CRL-1620), and SH-SY5Y neuroblastoma (ATCC-CRL-2266) cells. Before the experiment, the cells were cultured according to the supplier’s instructions. Briefly, neuroblastoma cells SH-SY5Y (ATCC-CRL-2266) were cultured in Dulbecco’s modified Eagle’s medium (DMEM)/Nutrient Mixture F-12 Ham (Sigma) supplemented with 10% (*v*/*v*) fetal bovine serum, penicillin (100 U/mL), and streptomycin sulfate (0.1 mg/mL). Human glioblastoma cell line A172 (ATCC-CRL-1620) and glioma cell line SW1088 (ATCC-HTB-12) were cultured in DMEM—high glucose (Sigma) supplemented with 10% (*v*/*v*) fetal bovine serum, 100 U/mL penicillin, and 0.1 mg/mL streptomycin sulfate. The culture media were renewed every three days, and the cells were passaged by trypsinization after reaching approximate confluency of 80%. 

### 2.2. Preparation of Cell Lysates

The medium from the attached cells was removed, and the cells were briefly washed twice with ice-cold Dulbecco’s phosphate-buffered saline (DPBS; Sigma). Subsequently, hypotonic ice-cold lysis buffer consisting of 50 mM TRIS with pH 7.5, supplemented with one mM ethylenediaminetetraacetic acid (EDTA; SERVA, Fisher Scientific, Göteborg, Sweden) and 1% (*w*/*v*) Triton X-100 (Sigma) was added to cells. The cells were scraped by using the rubber cell scraper. Lysates were transferred into the microfuge tubes and subsequently clarified by centrifugation at 10 000× *g* for 10 min. The supernatants were collected in aliquotes and either further analyzed or stored before use at −20 °C. 

### 2.3. Tumor Samples Processing and Dot-Blot Analysis

The Neurosurgery Department of Roosevelt Hospital in Banská Bystrica provided brain tissue samples obtained during tumor resection between 2015 and 2018. The current study included twenty biopsies from patients with glioblastoma multiforme, eight with meningioma, seven with astrocytoma, and four with oligodendroglioma (Table 1). Tissue samples were placed in RNAlater^®^ (Applied Biosystems/Ambion, Fisher Scientific, Göteborg, Sweden) right after surgery and stored at −80 °C. According to the manufacturer’s protocol, the extraction of protein from brain biopsies (25 mg of tissue) was performed using Isolate II RNA/DNA/protein Kit Phenol free (Bioline). The total protein was presented in the flow-through from the RNA Binding Step by column purification.

### 2.4. Estimation of Protein Concentration in Lysates

The protein level in lysates derived from cultured cells was estimated by the spectrophotometric method [38]. Bovine serum albumin dissolved in the lysis buffer was used as a protein standard. 

### 2.5. Western Blot

Twenty micrograms of lysate proteins, electrophoretically separated in 10% acrylamide gels by the SDS PAGE method [39], were transferred onto nitrocellulose membranes for Western blot analysis using Mini Trans-Blot cell (BioRad Laboratories, Bratislava, Slovakia). After blocking the unspecific binding sites on the membrane with blocking solution (BS1) consisting of Tris-buffered saline (TBS; 19.8 mM Tris, 136 mM NaCl, with pH adjusted to 7.5) supplemented 2% (*w*/*w*) albumin (BSA; Applichem), and 0.05% (*w*/*v*) Tween-20 (Sigma), the blots were incubated overnight with anti-MCC rabbit polyclonal (1:250) in BS1. After three washing steps with TBS supplemented with 0.05% (*w*/*v*) Tween-20 (TBS-T) solution, the blots were incubated in BS1 with secondary antibodies against rabbit IgGs covalently linked to horseradish peroxidase (1:10,000, Biorad Laboratories, Bratislava, Slovakia) for two hours at room temperature (RT). The SuperSignal West Pico Chemiluminescent Substrate (Thermo Fisher Scientific) solution was used to generate a chemiluminescent signal, which was visualized by the Chemidoc XRS system (BioRad Laboratories, Bratislava, Slovakia). The intensity of the MCC signal was normalized to the corresponding intensity of GAPDH signal as described [40]. We checked again in our imaging system if the figures of membranes were aligned with proper protein ladder. All three figures were obtained by using the same membrane. 

The putative discrepancies could be the consequence of the protein ladder figure is taken under visible light; therefore, there is a black part around the membrane, after that chemiluminescent signal of the first staining with anti-MCC/anti-rabbit IgG-POD was recorded, without light in dark, and this is automatically converted (therefore, the protein bands appear dark and membrane white). This is the “first round of figure recording”. Then, the membrane was removed from the imaging staining system chamber and restained with antibodies to detect GAPDH. After staining, we try to place the membrane on the same spot in the chamber for the “second recording round” of the chemiluminescent signal; this is performed manually, due to which some kind of the shift could be observed. Therefore, a slight rotation or shift between the protein standard figure, which was recorded in first round, and the signal of GAPDH (second round) may appear. However, the presence of the GAPDH signal serves as a positive control of WB methodology (protein loading, separation, electroblotting, binding to membrane, and appropriateness of chemiluminescent signal-generating solutions).

### 2.6. Dot-Blot Analysis

The extracted proteins from brain cancer samples were used to detect MCC expression by the dot blot method. The nitrocellulose membrane was spotted with 2 µg of tumor lysate protein. The membrane was incubated in BS1 at RT for 30 min to block the free binding sites on the membrane. Subsequently, the membrane was incubated with the mixture of rabbit antiserum against MCC diluted 1:250 in BS1 at 4 °C overnight. Afterwards, the membrane was washed three times with TBS-T. Subsequently, the membrane was probed with the solution of anti-rabbit IgG molecules conjugated with horseradish peroxidase (1:10,000) in BS1 for 2 h. The membrane was rinsed 3× in TBS-T for 10 min and 1× in TBS, and subsequently, the membrane was soaked in SuperSignal West Pico Chemiluminescent Substrate solution (Thermo Fisher Scientific). The chemiluminescent signal was recorded by the Chemidoc XRS system (BioRad Laboratories, Bratislava, Slovakia). The obtained chemiluminescent signals for MCC were quantified with the Image Studio Lite version 5.2 software (LI-COR Biotechnology).

### 2.7. Extraction of Biotin-Containing Proteins

Streptavidin-agarose beads (Thermo Fisher) were used for the extraction of biotin-containing proteins. The lysate from glioblastoma was diluted to the final concentration of 1 µg/mL protein in DPBS. Diluted lysate (150 µL) was mixed with 500 µL of streptavidin-agarose beads suspension and incubated at RT for one hour. Then, the suspension was centrifugated at 3000× *g* for 5 min, and the supernatant devoted to biotin-containing proteins was collected. Sediment was washed twice by resuspension in DPBS followed by centrifugation at 3000× *g* for 5 min. Supernatant generated during the washing step was collected and combined. Subsequently, the biotin-containing proteins were released from streptavidin-containing beads by incubation with biotin solution (100 µg/mL) in DPBS for 30 min. After incubation, the suspension was centrifuged at 3000× *g* for 5 min, and supernatant with biotin-containing proteins was collected. The proteins from all three fractions were, after precipitation with acetone, resuspended in DPBS and quantified with Bradford´s assay before further analysis for the presence of MCC by the dot-blot method.

### 2.8. Immunocytochemistry

The cells that were used for the immunocytochemical investigation were grown on sterile glass coverslips glued to the bottom in plates under standard conditions. When the cells reached approximately 50% confluency, the culture medium was discarded, and attached cells were briefly washed with ice-cold DPBS and subsequently fixed with paraformaldehyde (4% *w*/*v*; Sigma) solution in DPBS for 10 min. The fixation was stopped by the exchange of formaldehyde-containing solution by TBS. Before application of the primary antibodies, the fixed cells were permeabilized with TBS supplemented with 1% (*w*/*w*) Triton X-100 for 10 min and subsequently treated with blocking solution 2 (BS2) consisting of TBS and 1% (*w*/*v*) Triton X-100 supplemented with 2% (*w*/*v*) BSA at RT for 20 min. The primary antibodies, rabbit anti-MCC and human anti-PDH, were applied separately or together to obtain either a single or double labeling fluorescence signal. Both primary antibodies were diluted into a final ratio of 1:250 in BS2, and the antigen–antibody complex was allowed to develop overnight at 4 °C. The cells were washed three times with TBS supplemented with 1% (*w*/*w*) Triton X-100 before the mixture of the affinity-purified antibodies against rabbit or human IgG molecules was applied for 2 h. The anti-rabbit IgG molecules were covalently linked to Alexa Fluor 488 (Invitrogen, Thermo Fisher Scientific), while TRITC was bonded to anti-human IgG (Invitrogen, Thermo Fisher Scientific). Before mounting with the solidifying medium, the cells were washed three times in TBS. The cell nuclei were stained by adding DAPI (5 µg/mL) (Sigma) into the washing solution during the second-last washing step. Negative controls were prepared by omitting the primary antibodies.

### 2.9. Immunohistochemistry

Cryosections from human glioblastoma were prepared according to standard protocol. Frozen 15 μm thick sections were cut using the Shannon Cryotome E, Thermo Scientific Waltham, collected on SuperFrost Plus (VWR International, Radnor, PA, USA) glass microscope slides, and air-dried.

To minimize non-specific secondary antibody binding, the sections were first incubated in BS2 for 90 min at RT. Subsequently, samples were incubated with a mixture of primary antibodies: rabbit anti-MCC (1:250), human anti-PDH (1:250), and mouse anti-GFAP (1:250; Sigma) for overnight at 4 °C. Extensive washing 3 × 10 min in TBS supplemented with 1% (*w*/*w*) Triton X-100 was followed by the incubation with secondary antibodies: goat anti-rabbit linked with Alexa Fluor 488 (1:1000; Invitrogen), anti-human IgG conjugated with TRITC (1:1000; Invitrogen), and goat anti-mouse linked with Alexa Fluor 633 (1:1000, Invitrogen) for 90 min at RT. Slides were quickly rinsed with TBS, and subsequently, cell nuclei were co-stained by incubation in 5 µg/mL DAPI solution in TBS for 10 min at RT. Finally, slides were washed 3 × 10 min in TBS followed by a rapid rinse with H_2_O and mounted with VECTASHIELD^®^ Antifade Mounting Medium (Vector Laboratories).

### 2.10. Microscopic Analysis

The immunostained samples were imaged and analyzed using the Zeiss Axio Examiner/LSM 880 confocal system (Carl Zeiss, Jena, Germany). The 405 nm laser was used to visualize DAPI stained nuclei, the 561 nm laser to visualize TRITC-labeled PDH, and the 488 laser to visualize Alexa Fluor-488-labeled MCC. Individual samples were scanned using a Zeiss Plan-Apochromat 40×/1.3 Oil DIC M27 objective and Zeiss Zen Black software. Images were acquired with a resolution of 2048 × 2048 pixels, with the dwell pixel set to 2.06 μs and pinhole of 1.0 AU. For higher magnification images, the 2.0× zoom was used to investigate the details. The resulting images were processed using Zeiss Zen Blue software.

### 2.11. Enzymatic Estimation of 3-Hydroxybutyrate Release in Culture Medium

The cultured cells grown to approximately 80% of confluency were used to estimate the amount of released 3-hydroxybutyrate (3-OHB). At the beginning of the experiment, the culture medium was exchanged with the fresh DMEM/FBS, which contained leucine at a level of either 0.8 or 8.8 mM. After 24 h incubation, media were collected and used to estimate 3-OHB concentration, while the cells were lysed in a lysis buffer. The cell lysates were used for protein estimation. 

The concentration of 3-OHB in culture media was assessed by the enzymatic method [41]. Briefly, the proteins in collected culture media were thermally denatured at 100 °C for 5 min and removed by centrifugation at 10,000× *g* for 10 min. A total of 30 µL of clarified supernatant was mixed with 270 µL of reaction solution that consisted of 0.2 M glycine (Sigma)/0.13 M hydrazine (Sigma) buffer with pH 8.1 supplemented with 5 mM NAD^+^ (Roche, Basel, Switzerland) and 3 μg/mL of purified 3-hydroxybutyrate dehydrogenase from *Rhodobacter sphaeroides* (Roche). The generation of NADH was monitored spectrophotometrically at 340 nm by Synergy H4 microplate reader (Bio Tek, Winooski, VT, USA) until the absorbance reached the plateau lasting at least 5 min. The calibration curve, where the thermally deactivated medium was replaced by a solution of 3-OHB (Sigma) in DPBS, was used to assess the concentration of 3-OHB in culture media. Subsequently, the capability of cultured cells to release the 3-OHB was estimated by taking into account the molar amount of released 3-OHB into the culture medium by mg of cellular proteins for the 24 h duration of the incubation.

### 2.12. LC-MS Analysis

Culture medium from the cells, which were grown to 80% confluency, was exchanged to DPBS supplemented with 25 mM glucose and 2 mM leucine ^13^C_6,_ and cells were incubated for 4 h. Every hour during the incubation, an aliquot of the medium was sampled. The 40 μL of collected medium fractions were mixed with acetonitrile in a ratio of 1:10. The mixtures were subsequently centrifuged at 10,000× *g* for 10 min, and clarified supernatants were used for the LC-MS analysis. 

The polar compounds were separated on the Sequant ZiC-cHILIC 3 µm, 100 Å, 150 × 2.1 mm (MERCK) connected into a setup consisting of a liquid chromatography system LC NEXARA X2 (Shimadzu) coupled to mass spectrometer operating in IT-TOF mode (Shimadzu). For separation of internal standards and compounds in the medium, a mobile phase was used consisting of acetonitrile (eluent A; Sigma) and water with 100 mM ammonium acetate (eluent B; Sigma). The mobile phase was 10% B, which then increased to 60% over 20 min, afterward decreased back to 10% for the next 10 min. The flow rate was 500 µL/min. The obtained spectra were processed with Skyline 21.1 software (MacCross Lab Software).

### 2.13. ^1^H-NMR Experiment

Before the experiment, cells were grown up to 60% of confluency in 6-well plates under standard culture conditions. For an experiment, the standard culture medium was changed to a medium consisting of 90% (*v*/*v*) DMEM and 10% (*v*/*v*) FBS, and cells were incubated in a humidified incubator at 37 °C for 24 h. ^1^H-NMR analysis was performed on the collected clarified media following brief centrifugation. For analysis, 500 µL of the medium was enriched with 100 µL of NMR stock solution, which consisted of 0.5 M phosphate buffer pH 7.4, 0.2 mM sodium 3-(trimethylsilyl)propionic-2,2,3,3-D_4_ (TMS-D_4_). The mixture was transferred into glass NMR tubes with an inner diameter of 5 mm.

The NMR stock solution was prepared by dissolving its components in deuterated water. TMS-D_4_ was used as a chemical shift reference of the solution.

NMR spectroscopy of prepared mixtures was performed on Bruker Avance III 600 MHz, and the obtained spectra were solved using the human metabolomic database (www.hmdb.ca; access on 23 November 2021 Table 2), chenomics software free trial version, and internal metabolite database according to the previously described method [42]. 

The method of external standards was used to quantify the obtained signals.

### 2.14. Statistical Analysis

The mean ± standard error of the mean (SEM) of at least three independent experiments is expressed. The Student’s *t*-test tested the null hypothesis, and *p* < 0.05 was considered statistically significantly different.

## 3. Results

**Figure 1:**
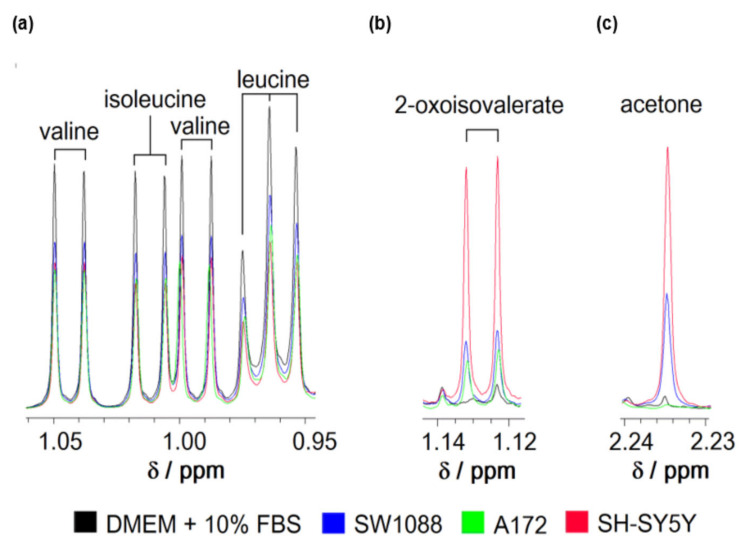
Representative spectra of branched-chain amino acids (leucine, isoleucine, and valine); (**a**), 2-oxoisovalerate (**b**) and acetone (**c**) of culture media consisting of DMEM supplemented with 10% FBS before (black) or after incubation of glioma (blue), glioblastoma (green), and neuroblastoma (red) cells for 24 h obtained by ^1^H-NMR spectroscopy. The ranges of chemical shifts (*δ*) with peaks for all three BCAAs (**a**); 2- oxoisovalerate (**b**) and acetone (**c**) are depicted.

To evaluate the capability of human cancer cells to metabolize leucine from their environment, the standard culture media from glioblastoma (A172), glioma (SW1088), and neuroblastoma (SH-SY5Y) cells was collected and subsequently subjected to analysis by a ^1^H-NMR method (Figure 1). The processing of the obtained ^1^H-NMR spectra revealed that all types of tested cells readily removed leucine, together with the remaining two BCAAs from their culture media (Figure 1a). The specific import rates of all three BCAAs (Table 3) were estimated after quantifying the level of BCAAs in culture media using external standards. The estimated specific import rates for BCAAs exceeded those for all other essential amino acids (data not shown). Simultaneously, the appearance of the peaks specific to 3-methyl-2-oxovalerate among obtained spectra indicates the capability of cultured cells to release BCKAs in their microenvironment (Figure 1b). The obtained ^1^H-NMR spectra did not allow the signal identification for two remaining BCKAs and their quantification. In addition, the signal for acetone could be recognized on the spectra with intensity increased in media after 24 h incubation (Figure 1c). 

**Figure 2:**
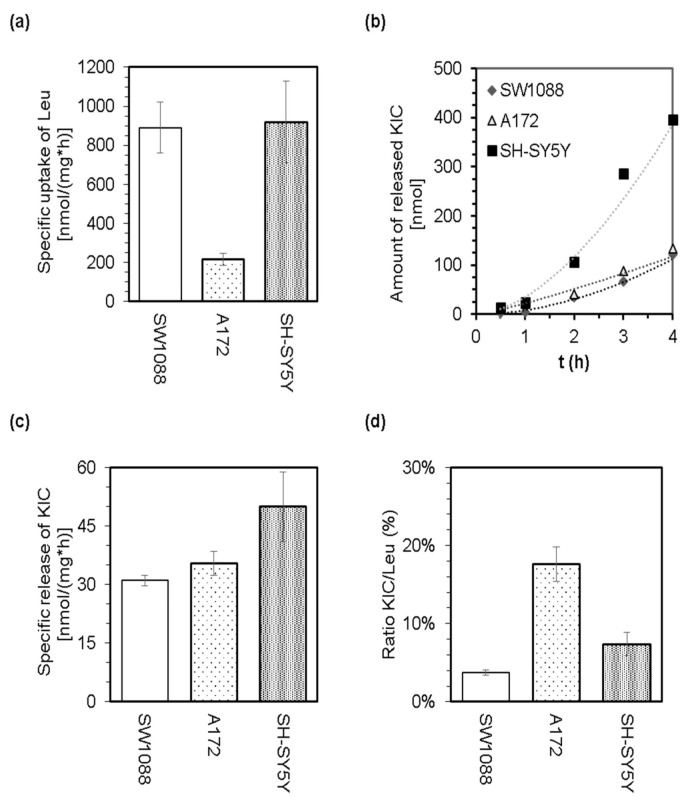
The capability of cultured glioma (SW1088), glioblastoma (A172), and neuroblastoma (SH-SY5Y) cells to withdraw leucine from culture media (minimal medium supplemented with 25 mM glucose and 0.8 mM leucine-^13^C_6_) and to release KIC. LC-MS analysis of the culture media allowed estimation of specific uptake of Leu (**a**), the time dependence of KIC release (**b**), the specific release of KIC (**c**), and calculation of the ratio of released KIC to withdrawn Leu (**d**).

The LC-MS analysis of culture media was employed to perform quantification of leucine disappearance from culture media (Figure 2a) and the simultaneous release of 2-oxoisocaproate (KIC) by cells (Figure 2b,c). The appearance of KIC in culture media (Figure 2b,c) demonstrates the capability of glioma, glioblastoma, and neuroblastoma cells to catalyze the prime step in the catabolic pathway of Leu. The calculated ratios for released KIC to disappeared Leu indicate that only a small proportion of the leucine molecules that disappeared from culture media are released in the form of KIC (Figure 2d). Therefore, it could be proposed that the majority of leucine molecules are further metabolized intracellularly. Since the cells were incubated in a minimal medium supplemented with 25 mM glucose and 0.8 mM leucine-^13^C_6_, but lacking other essential amino acids, we assume that leucine molecules might enter further catabolism instead of proteosynthesis.

**Figure 3:**
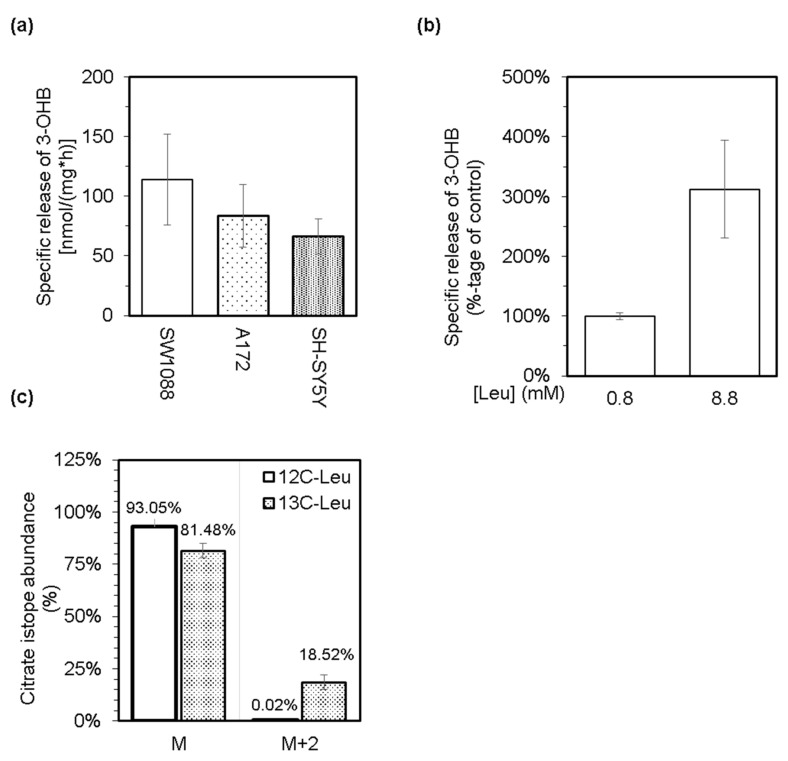
Levels of 3-hydroxybutyrate (3-OHB) in culture media from glioma (SW1088), glioblastoma (A172), and neuroblastoma (SH-SY5Y) cells were enzymatically estimated, and subsequently, the obtained values were used to calculate the specific release of 3-OHB (**a**). The effect of an increased level of leucine in culture medium (up to 8.8 mM) of neuroblastoma (SH-SY5Y) cells on their capacity to release 3-OHB (**b**). LC-MS was used to estimate the abundance of citrate isotopes released into the culture media from glioma cells (SW1088), which were incubated in a minimal medium supplemented with 25 mM glucose and either 0.8 mM leucine (12C-Leu) or 0.8 mM leucine-^13^C_6_ (13C-Leu; (**c**)). Group M represents the normal molar mass of citrate (191,124 g/mol), and group M + 2 represents the molar mass of citrate increased by two units (193,124 g/mol).

Based on the general knowledge that leucine is a purely ketogenic amino acid and can support the production of both ketone bodies, acetoacetate, and 3-hydroxybutyrate [26], we tested the possibility that cultured glioma, glioblastoma, and neuroblastoma cells might generate and release the ketone bodies into their media. Indeed, all three types of cultured cells possess the capability to generate and subsequently release acetone (Figure 1c) and 3-hydroxybutyrate (Figure 3a) into their milieu under standard culturing conditions. In addition, supplementation of a standard culture medium with leucine up to 8.8 mM level stimulated the release of 3-hydroxybutyrate from neuroblastoma cells (Figure 3b). This correlation between the increased level of leucine and the production of 3-hydroxybutyrate might indicate the capability of cancer cells to furnish the carbon atoms from leucine into their intermediary metabolism. Therefore, we cultured cells in a minimal medium supplemented with 25 mM glucose and 0.8 mM leucine-^13^C_6_. After 4 h of incubation, the media was analyzed by the LC-MS method for the ratio of citrate isotopes that are enriched with ^13^C atoms. Indeed, a substantially enriched fraction of citrate molecules with two units of molar mass heavier (M + 2) was detected (Figure 3c). This indicates that acetyl-^13^C_2_ moieties derived from leucine-^13^C_6_ could be incorporated into the structure of citrate by citrate synthase catalyzed reaction. In this respect, we assume that SW1088 cells can degrade leucine to acetyl-CoA and hence should express the appropriate enzymes. 

**Figure 4:**
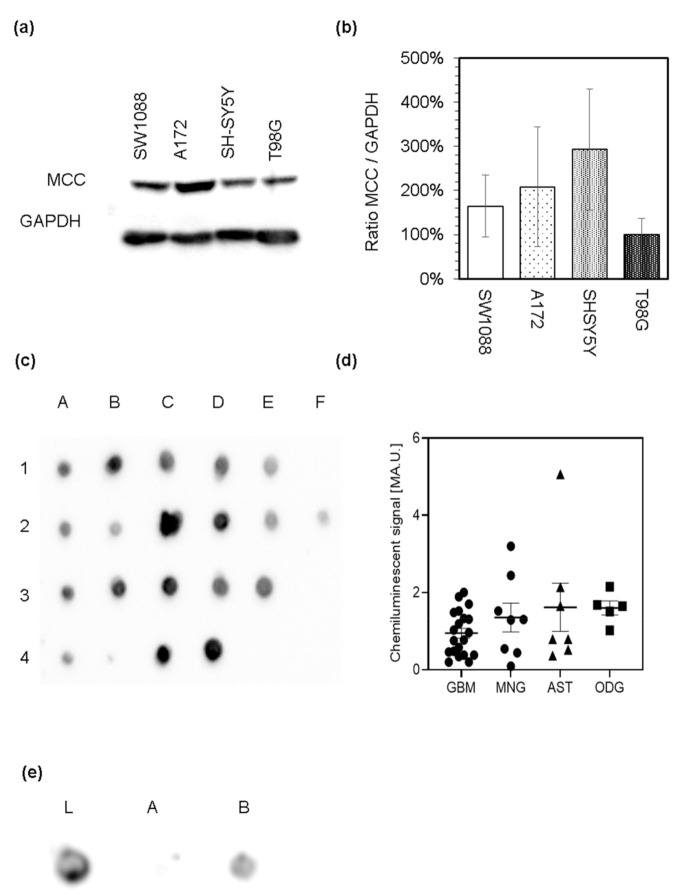
Immunoblotting analysis of the α subunit of 3-methylcrotonyl-CoA carboxylase (MCC) presence among the lysates derived from cultured human glioma (SW1088), glioblastoma (A172, T98G), and neuroblastoma (SH-SY5Y) cells (**a**). Relative expression levels of α subunit of MCC in different cell types was estimated by densitometry, using GAPDH as an internal standard (**b**)**.** Protein extracts derived from brain tumor samples were analyzed by the dot blot analysis for the presence of the α subunit of MCC. Protein extracts derived from glioblastoma multiforme samples (A1–E1), astrocytoma (A2–F2), oligodendroglioma (A3–E3), and meningioma (A4–D4) were spotted on the nitrocellulose membrane and probed with rabbit antiserum against MCC and subsequently with affinity-purified anti-rabbit IgG molecules covalently linked to horseradish peroxidase (**c**). Relative expression levels of MCC in different tumor lysates were estimated by densitometry (**d**). Protein extract derived from the glioblastoma multiforme sample was used to evaluate the specificity of the MCC signal. On the nitrocellulose membrane, proteins of glioblastoma lysate before (**e**; L) or after removal of biotin-containing proteins with streptavidin-agarose beads ((**e**); A) and also the proteins released from the complex with streptavidin after elution with biotin ((**e**); B) were spotted.

The capability of cells to catabolize leucine to acetyl-CoA strongly suggests that they possibly express the enzymes of leucine catabolic pathway. Out of the enzymes participating in leucine catabolism, only 3-methylcrotonyl-CoA carboxylase is considered specific for this pathway. Thus, we used the proteins in lysates derived from cultured glioma (SW1088), glioblastoma (A172), and neuroblastoma (SH-SY5Y) cell lines to evaluate the expression of MCC by immunoblotting analysis with the antibodies against MCC. Western blot analysis revealed the appearance of only one band (Figure 4a) with an estimated relative molecular mass of 75 kDa that corresponds to the mass of α subunit of MCC (Figure S1). Expression of α subunit of MCC among the used cells was normalized to chemiluminescent signal of glyceraldehyde 3-phosphate dehydrogenase (GAPDH; Figure 4a and Figure S1). Subsequent comparison of estimated relative quantities of MCCα showed that its expression does not differ among cultured cells (Figure 4b). Furthermore, the appearance of only one prominent band confirms the specificity of antibodies (Figure 4a and Figure S1).

**Figure 5:**
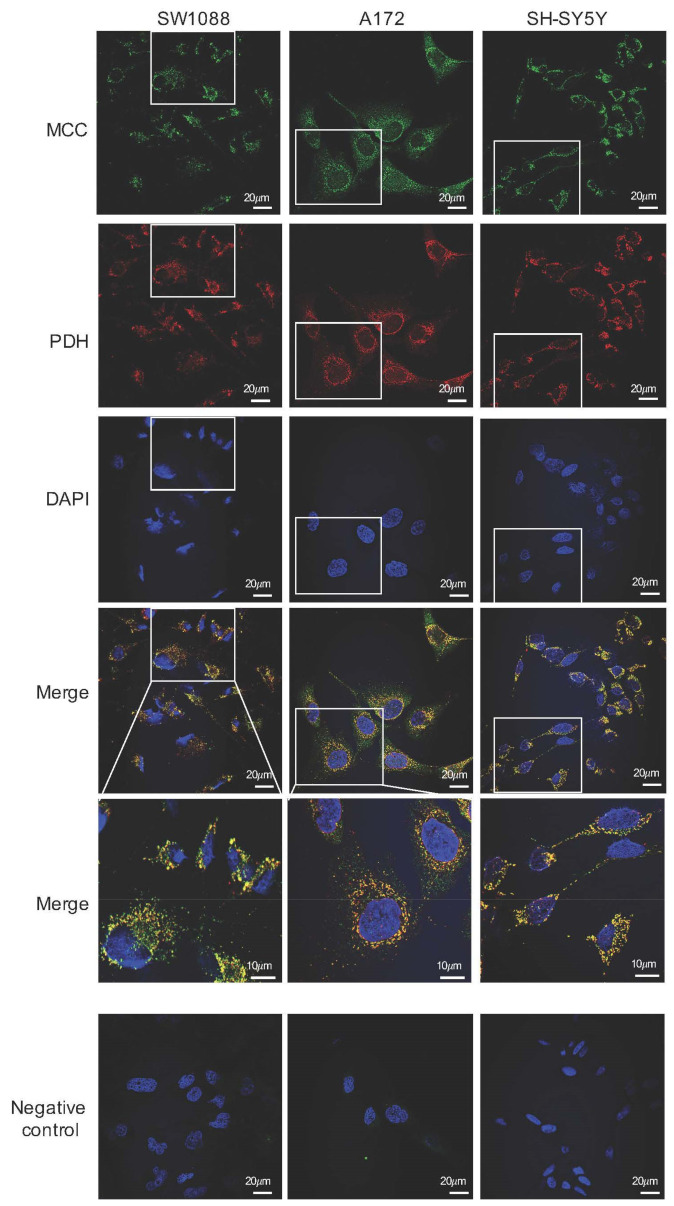
Immunofluorescence photomicrographs of human glioma (SW1088), glioblastoma (A172), and neuroblastoma (SH-SY5Y) cells, which were double-labeled with antibodies against the α subunit of 3-methylcrotonyl-CoA carboxylase (MCC; green) and pyruvate dehydrogenase (PDH; red). Cell nuclei were visualized by DAPI. The merged views are represented separately with higher magnifications (merge). Negative control was performed by omitting the primary antibodies (negative control). The scale bars represent either 20 or 10 μm.

**Figure 6:**
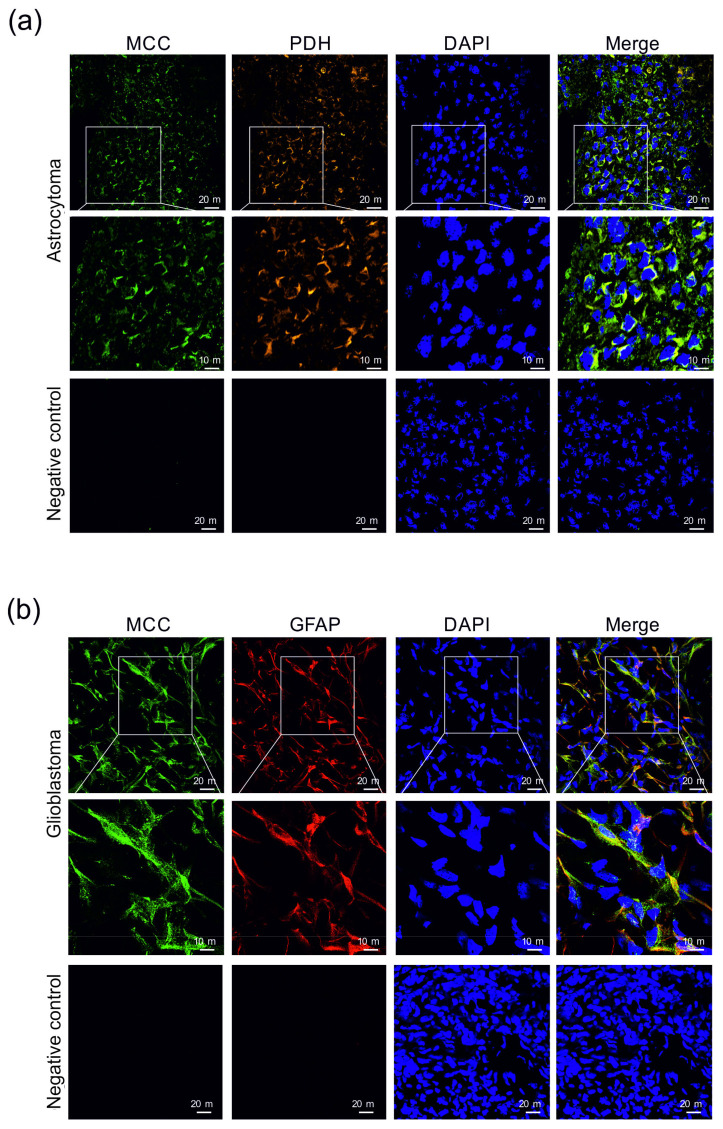
Immunohistochemical detection of the presence of 3-methylcrotonyl-CoA carboxylase (MCC) in human astrocytoma (**a**) and glioblastoma (**b**) sections. 3-methylcrotonyl-CoA carboxylase in the cells was immunofluorescently labeled with primary anti-MCC rabbit polyclonal antibodies followed by affinity-purified secondary antibodies conjugated to Alexa Fluor 488 (green). The presence of pyruvate dehydrogenase (PDH) among astrocytoma cells (**a**) was immunofluorescently labeled with primary anti-PDH human polyclonal antibodies followed by affinity-purified secondary antibodies conjugated to TRITC (orange). Mouse monoclonal antibodies were applied to detect GFAP (red) in cells of glioblastoma (**b**). Nuclei were visualized using DAPI intercalating fluorochrome (DAPI; blue). Merged views of the MCC, PDH, and DAPI are represented in the merge (yellow). Negative control was made by using secondary antibodies without primary antibodies (negative control). The scale bars represent either 20 or 10 μm.

The same antibodies were further used to investigate the presence of MCC in lysates (Figure 4c), cultured cells (Figure 5) and sections (Figure 6) derived from human brain tumors (Table 2). Surgically resected brain tumors samples were homogenized, and the expression of MCC was tested by dot blot analysis (Figure 4c,d). Obtained chemiluminescent signal from the qualitative analysis shows that MCC is present among all tested protein extracts obtained from astrocytoma, glioblastoma, meningioma, and oligodendroglioma (Figure 4c) tumors. To further confirm the dependence between MCC presence and appearance of a positive signal obtained by dot blot, the group of biotin-containing proteins was deprived of lysates by probing with streptavidin-agarose beads. Subsequent removal of biotin-containing proteins from the complex with streptavidin restored the signal appearance (Figure 4e). This result also highlights the specificity of used antibodies to recognize the biotin-containing MCC.

In combination with Alexa Fluor 488-conjugated secondary antibodies, polyclonal rabbit anti-MCC were also used for immunofluorescence detection of MCC expression in human neural cancer cell lines. Among all tested types of cells, namely glioma SW1088, glioblastoma A172, and neuroblastoma SH-SY5Y, a green fluorescence signal was visible when observed by fluorescence microscopy that was localized in the intracellular space with highest signal concentration near the cell nuclei (Figure 5). Indeed, polyclonal human anti-PDH combined with TRITC-conjugated secondary antibodies (red fluorescence signal) were used for immunofluorescence detection of a mitochondrial specific enzymatic complex of PDH (Figure 5). The colocalization of PDH (mitochondrial marker) with MCC in the same subcellular compartment could be confirmed by appearance of yellow color in merged views. Although the observed fluorescence signal was visible only when a combination of primary and secondary antibodies were used (Figure 5) and absent from samples where only secondary antibodies were applied (Figure 5), it can be assumed that the observed fluorescence signal corresponds with the presence of MCC in the cells.

Immunohistochemical analysis of astrocytoma and glioblastoma samples confirmed the presence of MCC in tumor-forming cells (Figure 6). In addition, the colocalization of MCC and PDH signals confirm the mitochondrial localization of MCC (Figure 6). 

## 4. Discussion

To evaluate the proficiency of human cancer cells in metabolizing leucine, we analyzed the presence of leucine and its metabolites in culture media from glioma, glioblastoma, and neuroblastoma cells by ^1^H-NMR and LC-MS methods. Analysis of the culture media by ^1^H-NMR method revealed that cultured glioma, glioblastoma, and neuroblastoma cells possess the capability to withdraw leucine from their microenvironment. Based on our obtained data, we estimated that the specific leucine uptake by all three cell types is approximately 20 nmol/(h ∗ mg of protein), which exceeded the uptake of two remaining BCAAs and other essential amino acids present in media. Transport of leucine through the plasma membrane is facilitated by several transporters and alterations in their expression and activity [15,16,43,44,45] promote leucine uptake during carcinogenesis [46,47].

Intracellular leucine is known to potentially assist in several essential signaling and metabolic functions [6,7,8,9,16,17,18,19]. As a signaling molecule, leucine possesses the potential to modulate the activity of elongation and initiation factors that are involved in the translation process [48,49,50], as well as mTOR signaling cascade [51,52,53,54]. Leucine contributes to several aspects of cellular metabolism and is an indispensable substrate for protein synthesis. Furthermore, leucine could contribute to nitrogen, anabolic, and energy metabolism by entering its catabolic pathway. The initial step of the leucine catabolism is reversible transamination to its cognate 2-oxo acid and 2-oxoisocaproic acid. Indeed, the appearance of 2-oxoisocaproic acid in culture media (Figure 2b,c) confirms that leucine undergoes its metabolic conversion by transamination. The reversible leucine transamination may be catalyzed by two isoforms of BCAT, BCAT1 or BCAT2, which possess the specific subcellular localization in either cytosol or mitochondria. Several studies confirmed the altered expression of both BCATs in animal and human cancer types or cells [10,11,23,55,56,57,58]. Both isoforms of BCAT reversibly transaminate leucine by transferring its amino group to 2-oxoglutarate by generating glutamate. This enzymatic conversion fuels the leucine-derived amino group into the cellular nitrogen metabolism and contributes to synthesizing non-essential amino acids and nucleotides in cancer cells [59]. A simultaneous decrease in the intracellular levels of 2-oxoglutarate may also affect the molecular mechanisms underlying the epigenetic regulation of gene expression [60], impacting the viability of cancer cells [11]. 

The release of 2-oxoisocaproate in culture media accounts for a minor part of leucine molecules disposal from cells as estimated by the LC-MS method (Figure 2). Therefore, we assume that a part of the 2-oxoisocaproate molecules might enter the cellular energy metabolism by passing through the BCKDH. BCKDH is a mitochondrial enzymatic complex that catalyzes the oxidative decarboxylation of all three branched-chain 2-oxo acids to their corresponding acyl-CoA derivatives. BCKDH is the main regulatory point for the entry of BCAAs in irreversible catabolism, and its activity is tightly regulated. The rat glioblastoma cells [24] and human colorectal [20] as well as pancreatic [21] cancer cells were shown to be equipped with the enzymatically active BCKDH. The passing of 2-oxoisovalerate through BCKDH initiates several-step conversion of the leucine-born carbon skeleton to acetoacetate and acetyl-CoA [17,21,24]. To test the capability of cultured human glioma, glioblastoma, and neuroblastoma cells in leucine metabolism, we estimated whether a reduced equivalent of acetoacetate, 3-hydroxybutyrate, is released into the culture media. Supplementation of the media with an increased amount of leucine enhanced cellular ketogenic capacity and led to the release of 3-hydroxybutyrate (Figure 3b).

In addition to leucine catabolism, several other metabolic cascades may participate in the production of ketone bodies. Therefore, to confirm the capability of cancer cells in catabolizing the leucine-derived carbon skeleton to common intermediates of cellular metabolism, we incubated cells in a minimal medium supplemented with leucine-^13^C_6_. Subsequent analysis of media revealed the appearance of several isomers of citrate, whose abundance in the media was quantified by the LC-MS method. The cells enriched their media with M + 2 isomer of citrate, which confirms the capability of cells to metabolize leucine into acetyl-CoA that enters the reactions of TCA cycle (Figure 3c).

Since the irreversible part of the leucine catabolism occurs in the mitochondrial compartment, catabolism of leucine generated acetyl-CoA could directly enter the intermediary metabolism of cancer cells. In this respect, leucine’s catabolism might be considered an alternative source of acetyl-CoA molecules for human glioma, glioblastoma, and neuroblastoma cells. Indeed, leucine was proven to supply acetyl moieties for fatty acid synthesis in adipocytes [61] and impact the metabolism of lipids in human pancreatic ductal adenocarcinoma [21]. In such a case, the leucine catabolism could be considered one of the alternative ways to counterpoise the suppressed production of acetyl-CoA through an inhibited PDH complex [62]. 

The capacity of cells to catabolize leucine into acetyl-CoA indicates that the cells are also equipped with the appropriate enzymes. These enzymes vary in their substrate specificity, and the majority of them are also common for metabolism of intermediates of the catabolism of other fatty acids. 3-methylcrotonyl-CoA carboxylase considered as an enzyme unique to the irreversible part of the leucine catabolic pathway [26]. MCC is a mitochondrial, biotin-containing enzyme, which consists of two subunits. Our immunocytochemical investigation confirmed the expression of MCC in mitochondrial compartments in tested cultured human glioma, glioblastoma, and neuroblastoma cells.

In addition, our results revealed that MCC is expressed in human astrocytoma, glioblastoma, meningioma, and oligodendroglioma tumors. Even though the number of analyzed tumor samples is limited, these results suggest that leucine can be a metabolic substrate for brain tumor cells. The expression of the MCC has also been confirmed previously in various breast [30], prostate [29,63], colorectal [31], and hepatocellular [32] tumors. Furthermore, several studies revealed novel signaling and regulatory functions intervened by the smaller subunit of MCC, MCCC2, in cancer cells [29,32]. In this respect, MCCC2 can affect the intracellular signaling cascade facilitated by promoting the activation of ERK in the cytosol of human hepatocarcinoma cells [32] or regulating the GLUD1-P38 MAPK signaling pathway in [29] prostate cancer cells. MCCC2 also exerts its regulatory function on cell proliferation and migration capabilities [29,30,31,32]. Presently, it remains unknown to which extent metabolic and regulatory roles of MCC in brain tumor-forming cells are critical. 

## 5. Conclusions

The expression of MCC by human cancer cells indicates their capability to employ leucine as a substrate for their metabolism. The catabolism of leucine could contribute to nitrogen metabolism of cancer cells and supply them with acetyl moieties that could subsequently enter the TCA cycle and support energy metabolism or be utilized as building blocks for anabolism of several biomolecules. 

## Figures and Tables

**Table 1:** Characteristics of the patients with brain tumors.

	Glioblastoma	Astrocytoma	Meningioma	Oligodendroglioma
Number	20	7	8	4
Age (years)	59 ± 11	33 ± 15	59 ± 8	49 ± 11
Gender (male/female)	11/9	4/3	3/5	1/3

**Table 2:** Chemical shifts (in ppm), J couplings (in Hz), and multiplicities for the pool of metabolites identified in cell culture medium by NMR. The ^1^H-NMR chemical shifts are reported relative to TMSP-D_4_ signal, to which the value of chemical shift equal to 0.000 ppm was assigned.

Metabolite	NMR Evaluation Range From–to	NMR Peak Assignment, Confirmed by Jres and Cosy, J–Coupling Constant (Hz)
Isoleucine	1.0124–1.027	0.941 (t ^1^; J = 7.48)
		1.0123 (d; J = 6.99)
		3.678 (d; J = 4.17)
Leucine	0.960–0.981	0.958 (d; J = 6.23)
		0.969d (d; J = 6.05) 1.679 (m) 1.720 (m) 1.749 (m)
Valine	1.030–1.065	0.9936 (d; J = 7.06) 1.044 (d; J = 7.06) 2.273(m)
		3.607 (d; J = 4.40)
2-Oxoisocaproate	2.600–2.628	0.943 (d; J = 6.63) 2.113(m)
		2.612 (d; J = 7.02)
2-Oxoisovalerate	1.126–1.137	1.11 (d; J = 7.05) 3.011(t)
3-Methyl-2-Oxovalerate	1.085–1.113	0.899 (t; J = 7.52) 1.104 (d; J = 6.74)
Acetone	2.231–2.239	2.235(s)

^1^ s: singlet; d: doublet; t: triplet; m: multiplet.

**Table 3:** Branched-chain amino acid removal capability of human glioma (SW1088; *n* = 6), glioblastoma (A172, *n* = 3), and neuroblastoma (SH-SY5Y, *n* = 6) cells from the culture media (DMEM/FBS). Concentrations of leucine, isoleucine, and valine in the media were estimated from ^1^H-NMR spectra by a method of external standards. Data are presented as the mean ± SEM.

Compound	Specific Uptake (nmol ∗ h^−1^ ∗ mg^−1^)					
	SW1088		A172		SH-SY5Y	
Leucine	26	±1	19	±3	23	±5
Isoleucine	25	±2	20	±2	22	±5
Valine	17	±2	15	±5	17	±5

## Data Availability

Data are available within the article or on request from the authors.

## Supplementary Materials

The following supporting information can be downloaded at: https://www.mdpi.com/article/10.3390/cancers14030585/s1, Figure S1. Original Images of Western Blot analysis of Figure 4a (right panel).
