# Supplementary material for: Expression of 3-Methylcrotonyl-CoA Carboxylase in Brain Tumors and Capability to Catabolize Leucine by Human Neural Cancer Cells
Source: Cancers (Basel). 2022 Jan 24;14(3):585. doi: 10.3390/cancers14030585 (PMC8833481; doi:10.3390/cancers14030585)
Supplement: Supplementary file 1 [file cancers-14-00585-s001.zip › cancers-1501639-supplementary-.pdf]

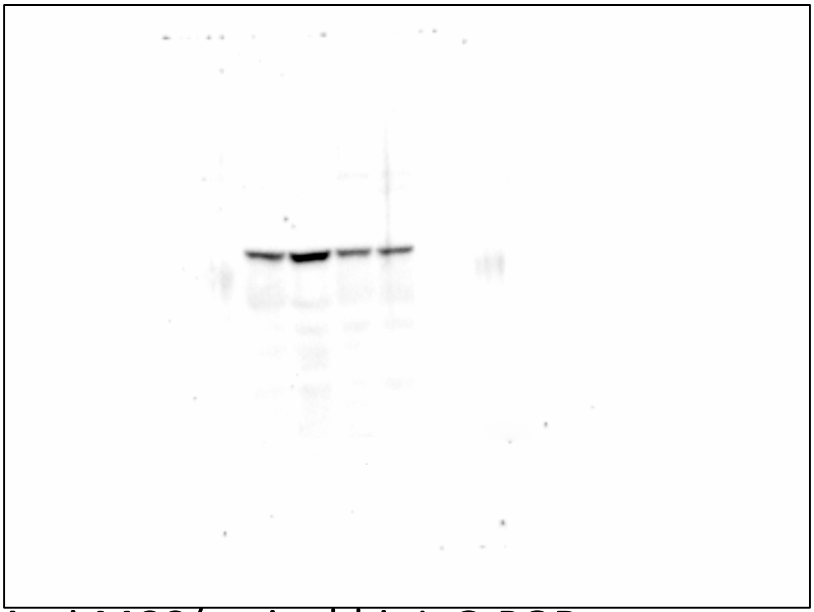

Anti-MCC/anti rabbit IgG-POD

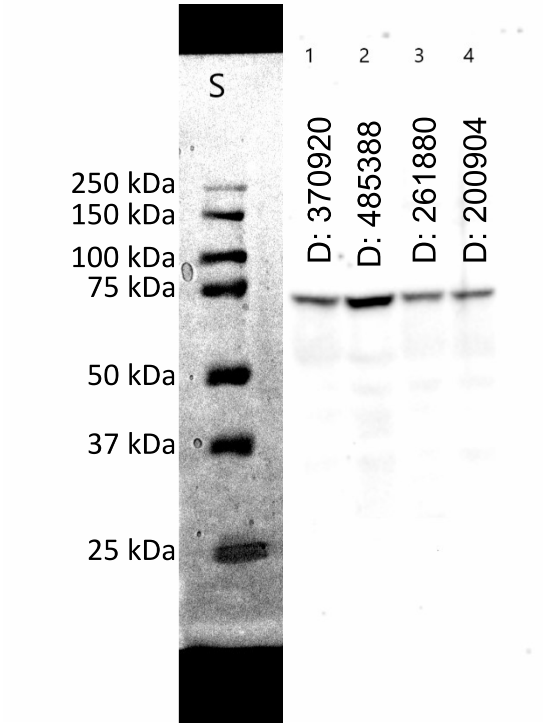

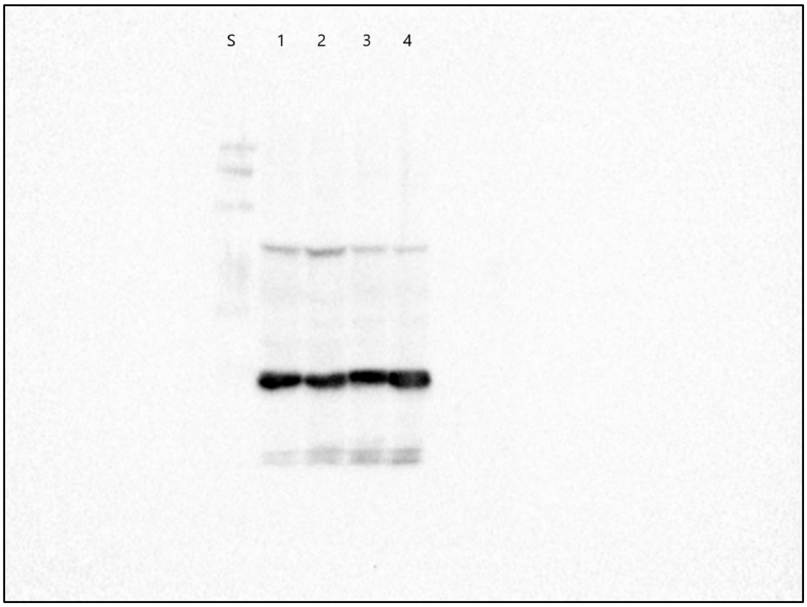

Anti-GAPD/anti mouse IgG-POD

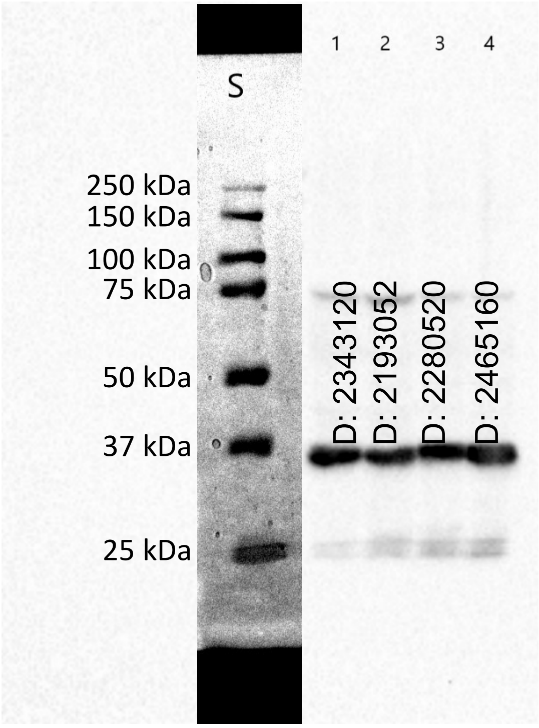

Figure S1: Original Images of Western Blot analysis of Fig. 4A (right panel). On the left, the protein ladder (S) was merged with protein standard and Mr of proteins added. Over the bands are the values of estimated density (**D:**) indicated. We checked again in our imaging system, if the figures of membranes were aligned with proper protein ladder. Indeed, all three figures were obtained by using the same membrane. The putative discrepances could be the consequence of: the protein ladder figure is taken under visible light, therefore there is a black part around the membrane, after that chemilumiscent signal of the first staning with anti-MCC/anti-rabbit IgG-POD was recorded - without light in dark and this is automatically converted (therefore the protein bands appear dark and membrane white). This is "first round of figure recording". Then membrane was removed from the imaging staining system chamber and restained with antibodies to detect GAPDH. After staining, even we try to place the membrane on the same spot in the chamber for "second recording round" of the chemiluminiscent signal, this is done manually a some kind of the shift could be observed. And therefore, slight rotation or shift between protein standard figure, which was recorded in first round, and signal of GAPDH (second round) may appear. However, the presence of GAPDH signal is there as a possitive control of WB methodology (protein loading, separation, electroblotting, binding to membrane, and appropriateness of chemiluniscent signal generating solutions).
